# Editorial: Advances in optical imaging for ophthalmology: new developments, clinical applications and perspectives

**DOI:** 10.3389/fopht.2024.1496015

**Published:** 2024-10-16

**Authors:** Pedro Mecê, Kiyoko Gocho, Wolf Harmening, Ethan Rossi, Laura Young

**Affiliations:** ^1^ Institut Langevin, ESPCI Paris, CNRS, PSL University, Paris, France; ^2^ Centre d’Investigation Clinique, Quinze-Vingts National Ophthalmology Hospital, Paris, France; ^3^ Rheinische Friedrich-Wilhelms-Universität Bonn, University Eye Hospital, Bonn, Germany; ^4^ Department of Ophthalmology, University of Pittsburgh School of Medicine, Pittsburgh, PA, United States; ^5^ Biosciences Institute, Newcastle University, Newcastle, United Kingdom

**Keywords:** ophthalmic imaging technology, optical coherence tomography, adaptive optics, optical imaging, image processing, clinical imaging applications

The field of ophthalmic optical imaging has undergone a profound transformation over the past three decades, particularly with the advent of optical coherence tomography (OCT) (1), which has since become the gold standard for a multitude of conditions. Significant advancements in both hardware and software have facilitated the emergence of numerous imaging techniques for increasingly high-resolution and high-contrast imaging of both the anterior and posterior eye. The latest generation of optical imaging modalities includes OCT angiography (OCTA) (2, 3), full-field OCT (4, 5), adaptive optics (AO) (6–8), phase contrast imaging (9–11), and functional imaging (12–15). These ophthalmic optical imaging modalities are being increasingly applied and translated into the clinical environment, where initial results indicate the potential for significant improvements in patient care. By elucidating the pathophysiological structures and functions of the eye’s intricate neurovascular network, these advances in imaging technology have the potential to facilitate earlier disease detection, more precise diagnosis and treatment monitoring, and more effective management of numerous ophthalmic diseases (16). In this Research Topic eight original research articles encompass a range of disciplines, including clinical imaging, image processing, multimodal image analysis, and functional retinal imaging. We offer here a concise overview of the entire Research Topic.

Five studies involved the evaluation and application of Adaptive Optics for clinical imaging and biomarker extraction at the micrometer scale. Among these five studies, two of them were focused on clinical imaging. Kempf et al. employed an AO fundus camera to examine the structure of the cone photoreceptor mosaic in the macula of eyes affected by retinitis pigmentosa (RP) related to Usher syndrome. A total of ten patients were enrolled in the study. The results demonstrated a reduction in cone density in patients with RP related to Usher syndrome, when compared to previously published data on healthy eyes. The authors emphasize that the AO-based high-resolution technique provides a valuable complement to established clinical examinations, offering deep phenotyping to identify patients for clinical trials as well as the potential to use this technique for treatment monitoring. The manuscript by Pedersen et al. aims to characterize retinal structural biomarkers for progression in adult-onset Stargardt disease from multimodal retinal imaging *in-vivo* maps. By using AO-SLO, the authors were able to identify dark cones that were not visible in other modalities, such as OCT and fundus photography. The presence of dark cones may indicate the initial stages of retinal disease progression in adult-onset Stargardt disease. Two other studies focused on studying and establishing reference values to refine quantitative biomarkers, enabling comparability accross different studies. Warr et al. investigates how the size of the sampling window affects topographical mapping of foveal cone density using AO scanning light ophthalmoscope images. The study included 44 participants with normal vision and analyzed 440 foveal cone density maps created with varying window sizes (5 to 200 cones). Key metrics, such as peak cone density (PCD) and cone density centroid (CDC), were compared across different window sizes. Overall, this study underscores the importance of sampling window size in the assessment of foveal cone density, highlighting that CDC metrics offer more consistent results compared to PCD metrics. Understanding these variations is crucial for improving the comparability of cone density data across different studies. Kortuem et al. address a gap in the available dataset by utilizing an AO fundus camera to establish reference values for the wall-to-lumen ratio (WLR) across different age groups and at varying retinal locations. By imaging 50 right eyes of healthy individuals, the authors were able to establish normative values for five distinct age groups. Their findings show no significant differences between the age groups, and neither were there any significant impacts from normotensive blood pressure parameters. Finally, the authors highlight that AO-based vessel analysis may provide clinically useful biomarkers for cardiovascular health and should be tested in future studies. Finally, Kalitzeos et al. focused on an image processing method to enhance image quality. Using images generated with AO-SLO in a quadrant detection scheme, they propose the use of emboss filtering and minimum intensity projection as an image processing pipeline to enhance the visualization of photoreceptor cells. The proposed method allows for the generation of enhanced images of the photoreceptor mosaic, thereby facilitating the identification of individual cells through the application of straightforward image processing techniques.

Besides analysis of retinal structure, a study from Pfäffle et al. proposes to probe retinal function. They present a method for distinguishing between the functional signals of rod and cone photoreceptors in the human retina using full-field swept-source optical coherence tomography (FF-SS-OCT). To this end, the authors employed a mathematical model that enables the separation of rod and cone responses based on their distinct temporal dynamics. This approach represents a significant advancement in functional retinal imaging, offering insights into the separate contributions of rods and cones without the necessity for high-resolution spatial separation. Finally, two reviews discuss and analyze the field of ophthalmic imaging. Meng et al. provide an overview of both overt manifestations and subtle structural changes of the ocular fundus, specifically within the retina and the choroid, among individuals with systemic lupus erythematosus (SLE). The authors illustrate how recent advances in multimodal ophthalmic imaging have enabled ophthalmologists to detect subclinical microvascular and structural changes in the fundus of patients with SLE who do not present with ocular manifestations. Zhang et al. provides a comprehensive overview of techniques aimed at enhancing the quality of retinal fundus images. Suboptimal images are frequently produced due to factors such as inadequate illumination, scattering, or blurriness. The review introduces various computational methods for image restoration, including illumination correction, dehazing, and deblurring. Furthermore, the review addresses deep learning methodologies to enhance retinal images by learning from synthetic degraded images.

The Guest Editors would like to express their sincerest gratitude to all of the authors and the numerous reviewers who contributed to this Research Topic. Furthermore, the editors would like to express their appreciation for the dedicated Frontiers in Ophthalmology staff, whose consistent professionalism and patient support were instrumental in the success of this undertaking.
